# Regenerating Lost Muscle: *Msx1* to the Rescue

**DOI:** 10.1371/journal.pbio.0020266

**Published:** 2004-08-17

Cell and molecular biologists have a good start at understanding the adult salamander's enviable ability to completely regrow a lost limb or jaw. (Salamanders can even regenerate portions of their eyes and heart.) Happily, mammals share many of the required cellular skills—though in an untapped form. In response to injury, fully differentiated salamander muscle cells can produce less specialized cells that are capable of multiplying and recreating lost tissue. In this month's *PLoS Biology*, biochemist Jeremy Brockes and his colleagues at University College London tie this muscle cell de-differentiation to expression of a single gene, *Msx1*, a player in limb development and regeneration across species.[Fig pbio-0020266-g001]

**Figure pbio-0020266-g001:**
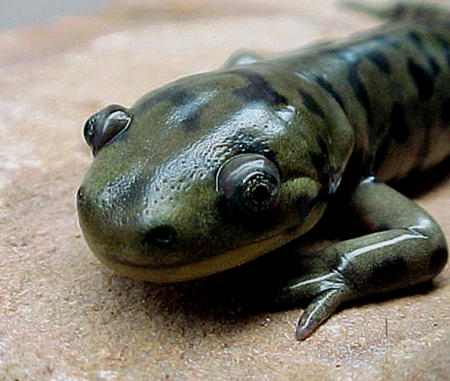
An adult salamander

The protein encoded by *Msx1* appears at the borders of maturing tissues in salamanders, chickens, and mice, and represses differentiation of muscle cells (called myofibers) during development. *Msx1* promotes amphibian limb and tail regeneration, zebrafish fin repair, and regrowth of the tips of digits in mice. Here, Anoop Kumar et al. clarify the gene's role in muscle repair by demonstrating that *Msx1* expression is required for de-differentiation of salamander myofibers—mature muscle tissue without the ability to produce new cells—into cells with single nuclei (mononucleate cells) that are able to multiply.

Large, elongated cells, myofibers are distinguished by a striped pattern of actin and myosin, the proteins that produce muscle contractions. They develop from precursor cells, which proliferate and fuse together into multinucleate intermediates called myotubes. As myotubes bulk up on actin and myosin, pushing their many nuclei to the cell periphery, they mature into myofibers. Once differentiated, myofibers are committed to the life of a muscle workhorse. They cannot divide; mature muscle in mammals adds strength and repairs injury by accumulating more actin and myosin, and by fusing single-nucleus “satellite” cells into new or existing myofibers.

Unlike their mammalian counterparts, salamander and newt myofibers respond to injury by splitting into several multinucleated fragments, or by budding off several of their nuclei to create individual cells. These mononucleate progeny multiply and develop to replace lost tissue. Kumar et al. found that more than half of salamander myofibers spontaneously fragment and bud when dissected and placed in cell culture. Prior study has shown that mouse myofibers don't normally behave this way, although cultured mouse myotubes can imitate amphibian myofibers if given a push, in the form of *Msx1*. A fraction of mouse myotubes made to express *Msx1* break off mononucleate cells that can be induced to express markers for bone, cartilage, fat, or muscle.

In the salamander cells, *Msx1* RNA and protein appeared in actively fragmenting and budding myofibers, especially in and around their nuclei. When Kumar et al. blocked the translation of *Msx1* mRNA, the myofibers did not generate new cells.

The results confirm *Msx1* as a pivotal regulator of muscle cell re-entry into the cycle of cell division and tissue growth. In this context, it doesn't drive cell division—on the contrary, the authors showed that *Msx1* induced cell splitting without DNA replication. The gene drove committed muscle cells to donate nuclei, creating a pool of new cells to divide, differentiate, and repair damage. Given what it can do for mouse cells in culture, the question remains whether *Msx1* might help awaken a latent capacity for regeneration in living mammals.

